# Mu-opioid receptor-dependent transformation of respiratory motor pattern in neonates *in vitro*

**DOI:** 10.3389/fphys.2022.921466

**Published:** 2022-07-22

**Authors:** Maia G. Gumnit, Jyoti J. Watters, Tracy L. Baker, Sarah M. Johnson, Stephen M. Johnson

**Affiliations:** Department of Comparative Biosciences, School of Veterinary Medicine, University of Wisconsin-Madison, Madison, WI, United States

**Keywords:** mu-opiod receptor, neuromodulation, respiratory motor control, neonatal rat, *in vitro*

## Abstract

Endogenous opioid peptides activating mu-opioid receptors (MORs) are part of an intricate neuromodulatory system that coordinates and optimizes respiratory motor output to maintain blood-gas homeostasis. MOR activation is typically associated with respiratory depression but also has excitatory effects on breathing and respiratory neurons. We hypothesized that low level MOR activation induces excitatory effects on the respiratory motor pattern. Thus, low concentrations of an MOR agonist drug (DAMGO, 10–200 nM) were bath-applied to neonatal rat brainstem-spinal cord preparations while recording inspiratory-related motor output on cervical spinal roots (C4-C5). Bath-applied DAMGO (50–200 nM) increased inspiratory motor burst amplitude by 40–60% during (and shortly following) drug application with decreased burst frequency and minute activity. Reciprocal changes in inspiratory burst amplitude and frequency were balanced such that 20 min after DAMGO (50–200 nM) application, minute activity was unaltered compared to pre-DAMGO levels. The DAMGO-induced inspiratory burst amplitude increase did not require crossed cervical spinal pathways, was expressed on thoracic ventral spinal roots (T4-T8) and remained unaltered by riluzole pretreatment (blocks persistent sodium currents associated with gasping). Split-bath experiments showed that the inspiratory burst amplitude increase was induced only when DAMGO was bath-applied to the brainstem and not the spinal cord. Thus, MOR activation in neonates induces a respiratory burst amplitude increase *via* brainstem-specific mechanisms. The burst amplitude increase counteracts the expected MOR-dependent frequency depression and may represent a new mechanism by which MOR activation influences respiratory motor output.

## Introduction

Opioid drugs that activate mu-opioid receptors (MORs) are widely used to provide analgesia but are also commonly misused due to their addictive properties. A major negative clinical side effect of MOR drugs is respiratory depression because effective analgesic doses are up to ×100 greater than the doses that inhibit breathing (Czapla et al., 2000). The neuronal mechanisms by which MOR drugs cause respiratory depression are under intense investigation given their associated mortality and morbidity (Palkovic et al., 2020; Bateman and Levitt, 2021; Ramirez et al., 2021). MOR-induced respiratory depression involves several respiratory-related regions in the pons and medulla including the preBotzinger complex, parabrachial nucleus/Kolliker-Fuse complex, and premotor neurons in the caudal ventral respiratory group (Stucke et al., 2008; Montandon and Horner, 2014; Bachmutsky et al., 2020; Palkovic et al., 2020; Saunders and Levitt, 2020; Palkovic et al., 2021). However, respiratory neurons in these regions are also modulated by many neurotransmitters including endogenous opioid peptides that activate MORs (Doi and Ramirez, 2008). Breathing is a highly complex, integrated, and state-dependent motor output (Doi and Ramirez, 2008), and the physiological reasons why respiratory-rhythm generating neurons express MORs are not well understood.

Most studies on the impact of MOR activation on breathing focus on inhibitory effects on breathing, but MOR activation can also result in a combination of inhibitory and excitatory effects. Intravenous infusion of DAMGO induces a short-lasting ventilatory depression, followed by a 10–12 min ventilatory increase in adult rats (Czapla et al., 2000). Likewise, DAMGO infusion into young arterially perfused, decerebrate rats decreases phrenic motor output amplitude but increases breathing frequency (Koganezawa et al., 2011). In neonatal rat *in vitro* preparations that spontaneously produce respiratory-related motor output, bath-applied DAMGO decreases motor burst frequency, but increases motor burst amplitude in hypoglossal nerve roots (Johnson et al., 1996) and cervical spinal roots (Barnes et al., 2007). In addition, bath-applied DAMGO increases fictive breathing frequency in neonatal rat pons-attached brainstem-spinal cord preparations (Tanabe et al., 2005). It is possible that respiratory depression observed at higher opioid drug doses obscures the complex neuromodulatory effects of lower doses. Since the MOR-dependent increase in inspiratory-related motor burst amplitude is not well understood nor a universal finding, we chose to investigate this phenomenon further at low DAMGO concentrations. Characterizing changes in respiratory motor output due to low level MOR activation may lead to new hypotheses regarding the function of endogenously released opioid peptides.

To address this question, we used rhythmically-active brainstem-spinal cord preparations from neonatal rats to ask whether: (1) MOR activation with low DAMGO concentrations increases inspiratory motor burst amplitude; (2) persistent inspiratory burst amplitude increase induced by DAMGO represents a form of neuroplasticity; (3) crossed spinal pathways within the phrenic motor nucleus contribute to the DAMGO-induced inspiratory burst amplitude increase; (4) the DAMGO-induced inspiratory burst amplitude increase is expressed in thoracic motoneuron pools; (5) the DAMGO-induced inspiratory burst amplitude increase requires activation of persistent sodium currents similar to gasping *in vitro*; (6) the DAMGO-induced inspiratory burst amplitude increase is due to MOR activation in the spinal cord or brainstem.

## Methods

### Neonatal rat brainstem-spinal cord preparations

All experimental procedures followed NIH guidelines and this study was approved by the University of Wisconsin-Madison Institutional Animal Care and Use Committee. The sex of neonatal (P0-P3) Sprague-Dawley rats (Charles River, Wilmington, MA, United States) was determined by visual inspection of the urogenital distance. Neonatal rat pups were anesthetized with 5% isoflurane (balance O_2_) before decerebration. The remaining tissue was placed in ice-cold artificial cerebrospinal fluid (aCSF), composed of (in mM): 120 NaCl, 26 NaHCO_3_, 20 glucose, 2 MgSO_4_, 1 CaCl_2_, 3 KCl, and 1.25 Na_2_HPO_4_. All dissections were performed in ice-cold aCSF before transferring the preparations to a standard recording chamber (volume = 0.75 ml) where they were continuously bathed with oxygenated aCSF solution (26°C, aerated with 5% CO_2_ and 95% O_2_, pH = ∼7.4) at a flow rate of 4–5 ml/min.

**FIGURE 1:**
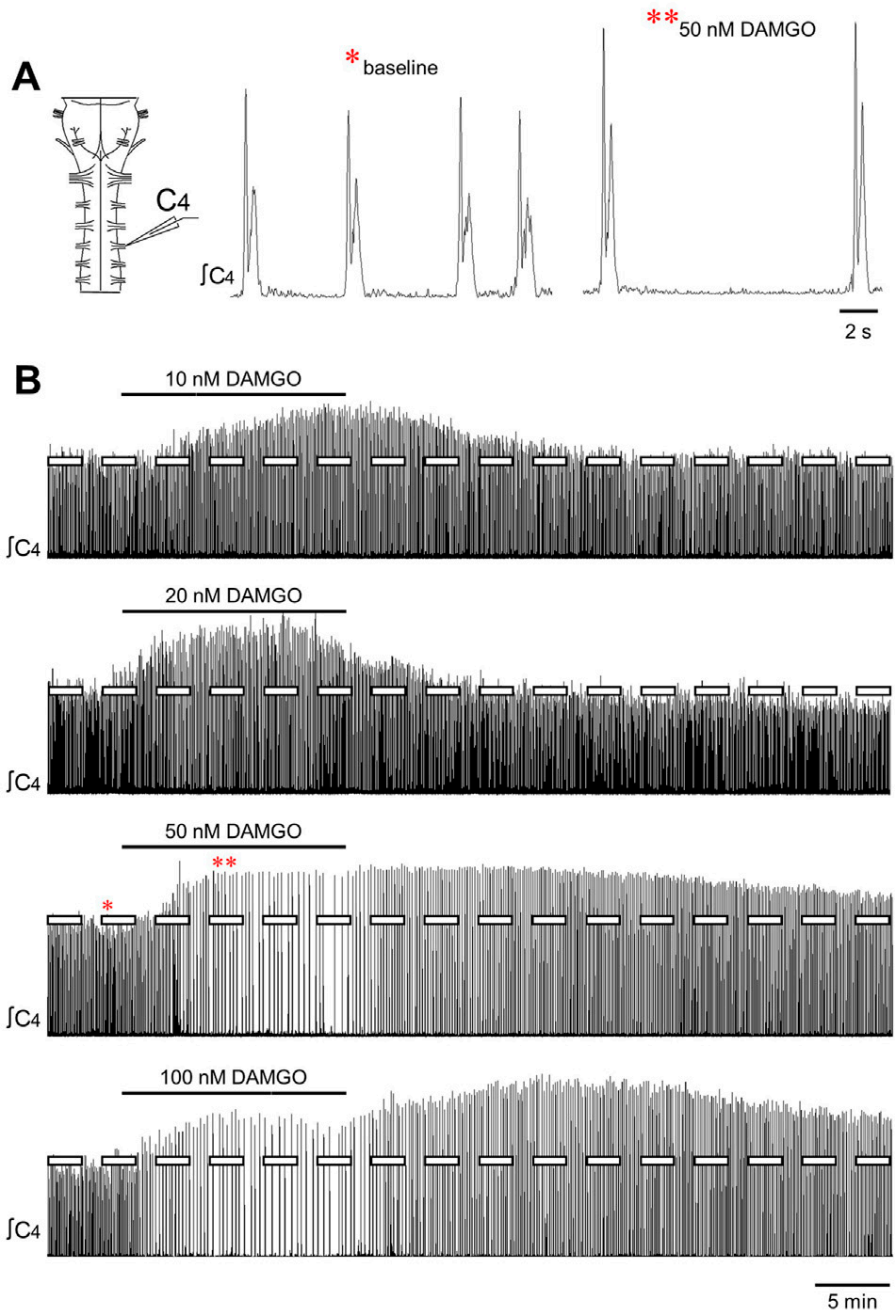
DAMGO produced changes in inspiratory-related motor output *in vitro*. **(A)** Drawing of a brainstem-spinal cord preparation with suction electrode attached at ventral cervical spinal root C4. At right is a sample voltage trace showing inspiratory-related motor bursts. **(B)** Effects of bath-applied DAMGO on inspiratory-related motor output are shown. DAMGO typically increased burst amplitude and decreased burst frequency.

Several different types of brainstem-spinal cord preparations were used in this study. The brainstem-spinal cord preparation most commonly used was cut slightly rostral to the level of cranial nerve IX (Figure 1A) and caudally at the level of cervical spinal segment C7. The parafacial/retrotrapezoid nucleus (pFRG/RTN) was likely intact and included in these preparations (see Figure 1 in Ballanyi et al., 2009). In other brainstem-spinal cord preparations, the rostral cut was the same, but the spinal cord was cut along the midline from C3 to C7 and the segments were pinned slightly away from each other (Figure 5A). This “midline-lesioned” spinal cord modification disrupted crossed pathways at these cervical levels (Zimmer and Goshgarian, 2005). To test whether thoracic spinal motor output was altered by drug application, brainstem-spinal cord preparations were cut rostrally as described above, but the caudal cut was at thoracic spinal segment T4 or T8 (Figure 6A). For some brainstem-spinal cord preparations, a “split-bath” configuration was established whereby the recording chamber was split into two compartments (e.g., brainstem and spinal cord) with a plastic barrier and petroleum jelly at the level of C2 that allowed brainstem and spinal cord to be bathed with different aCSF solutions (Figure 8A). It should be noted that brainstem-spinal cord preparations lack chemo- and mechanosensory feedback mechanisms that work together to maintain arterial blood-gas homeostasis by altering respiratory motor pattern.

All brainstem-spinal cord preparations were allowed to equilibrate for 30–65 min before recording baseline data and initiating experimental protocols. Some preparations were not exposed to any drug to assess time-dependent changes in inspiratory motor output; these are referred to as “time controls.” All other preparations were exposed to only one drug concentration or drug combination (e.g., DAMGO followed by naloxone, DAMGO during a background riluzole concentration). Spontaneously produced inspiratory-related motor output was recorded by attaching glass suction electrodes to ventral cervical (C4-C5; includes phrenic motoneurons) or thoracic (T3-T7; includes intercostal motoneurons) nerve roots. In most preparations, motor output was recorded bilaterally at C4-C5. Signals were acquired at 50 Hz, amplified (10,000x) and band-pass filtered (0.1–500 Hz) using a differential AC amplifier (model 1700, A-M Systems, Everett, WA, United States) before being rectified and integrated (time constant = 50 ms) using a moving averager (MA-821/RSP, CWE, Inc., Ardmore, PA, United States). Data were collected using Axoscope hardware and software (Molecular Devices, Sunnyvale, CA, United States).

### Experimental drugs and chemicals

DAMGO (MOR agonist; [D-Ala2, N-Me-Phe4, Gly5-ol]-Enkephalin acetate salt) was obtained from Sigma-Aldrich (St. Louis, MO, United States), naloxone was obtained from Santa Cruz Biotechnology (Dallas, TX, United States), and riluzole was obtained from VWR International (Radnor, PA, United States). DAMGO and naloxone were dissolved in water and frozen into aliquots. Riluzole was dissolved in dimethylsulfoxide (DMSO) and frozen into aliquots.

### Statistical analysis

**FIGURE 2:**
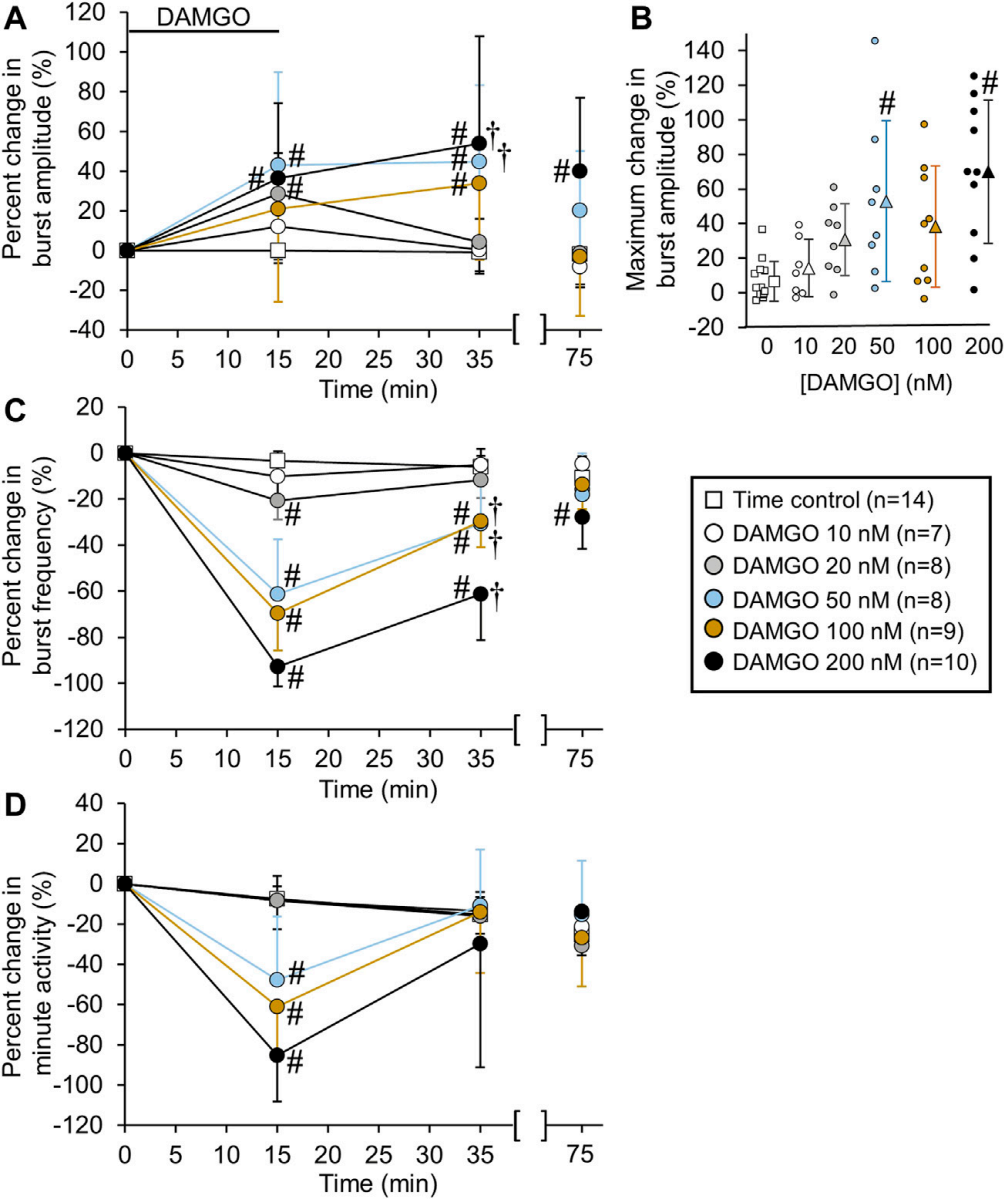
DAMGO increased inspiratory-related motor burst amplitude and decreased burst frequency. **(A)** Percent change in burst amplitude relative to baseline for untreated time controls (white squares) and preparations exposed to DAMGO at 10 nM (white circles), 20 nM (gray circles), 50 nM (blue circles), 100 nM (brown circles), and 200 nM (black circles). The 15-min time point represents the steady-state response after a 15-min DAMGO application, and the 35-min time point represents the time after 20 min of washout. The 75-min time point represents the time after 60 min of washout. **(B)** The maximum percent change in burst amplitude is shown for the time controls and each DAMGO concentration. The maximum percent change in burst amplitude occurred during DAMGO application or the washout period. The circles represent individual experiments and the triangles (following the same color scheme as the circles for the DAMGO concentrations) represent the mean maximum percent change in burst amplitude. **(C)** Burst frequency was decreased at nearly all DAMGO concentrations and returned to near baseline levels after 60 min of washout. **(D)** Minute activity decreased during DAMGO application but returned to near time control levels at the 35-min time point. The pound sign (#) indicates a significant pairwise comparison difference from time controls at that time point, and the dagger (†) indicates a significant overall DAMGO concentration effect. Data shown as mean ± SD.

**FIGURE 3:**
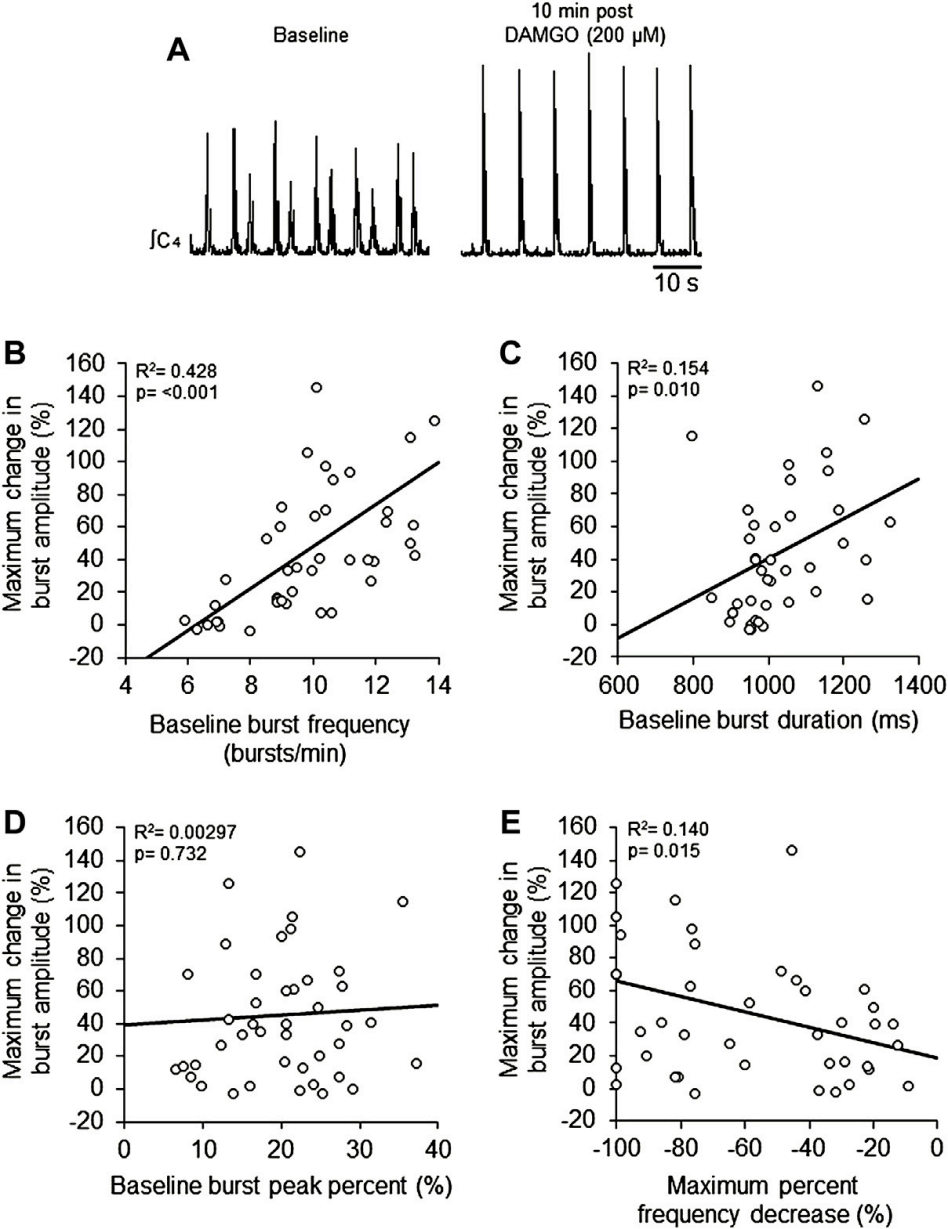
DAMGO-induced inspiratory burst amplitude increase was dependent on baseline properties of inspiratory motor burst output. **(A)** Voltage traces of inspiratory motor output from a brainstem-spinal cord preparation with a relatively high baseline burst frequency (left), and the large burst amplitude increase at 10-min post-DAMGO administration (right). Linear regression analysis of maximum change in burst amplitude were compared to: **(B)** baseline burst frequency, **(C)** baseline burst duration, **(D)** baseline burst peak percent, and **(E)** maximum percent decrease caused by DAMGO applications. Preparations with relatively higher baseline burst frequencies (B) and longer burst durations (C) exhibited larger DAMGO-induced increases in inspiratory burst amplitude. DAMGO-induced increases in inspiratory burst amplitude were inversely proportional to the magnitude of burst frequency decreases during DAMGO application (E).

Voltage traces of spinal inspiratory-related motor output or “bursts” were analyzed using Clampfit software (Molecular Devices, Sunnyvale, CA, United States). For most experiments, inspiratory motor burst variables from left and right cervical ventral roots were averaged for each preparation. For preparations that included the thoracic spinal cord, only one cervical and thoracic ventral root recording was analyzed. Burst amplitude was measured at the peak of integrated nerve discharge in arbitrary units and normalized to baseline. Burst frequency was measured as bursts/min, burst duration was the duration of the bursts (ms), and burst rise time was the time (ms) from 10% to 90% of the initial increase of the burst to the peak discharge. Burst “peak percent” was calculated as the (rise time)/(burst duration) to estimate the percent of the burst duration when the peak discharge occurred. Minute activity was the sum of the integrated motor bursts in arbitrary units within 5-min bins and normalized to baseline. Data were averaged into 5-min bins, expressed as the percent change from baseline values, and reported as mean ± SD. In most cases, a two-way repeated measures ANOVA (time and DAMGO concentration were the two factors) was performed with post-hoc comparisons using the Student-Newman-Keuls test in SigmaStat software (Jandel Scientific Software, San Rafael, CA, United States). A one-way ANOVA was performed for the data in Figure 2B while linear regression was performed for data in Figure 3. Statistical significance was accepted when *p* < 0.05.

## Results

### DAMGO increased inspiratory burst amplitude and decreased burst frequency

Bath-applied DAMGO (10–200 nM, 15 min) increased inspiratory-related motor burst amplitude and decreased burst frequency (Figures 1A,B). In some experiments in which 100 or 200 nM DAMGO was applied, burst amplitude also increased during the washout period before returning back to baseline levels. Accordingly, drug- and time-dependent changes in inspiratory burst variables were quantified at both the 15-min (steady-state DAMGO) and 35-min (after 20 min of washout) time points and described as percent change from baseline (Figure 2). Brainstem-spinal cords were either untreated (time controls; *n* = 14) or exposed to DAMGO at 10 nM (*n* = 7), 20 nM (*n* = 8), 50 nM (*n* = 8), 100 nM (n = 9), or 200 nM (n = 10). Burst amplitude increased compared to time controls at the end of the DAMGO application (15-min time point) by 29 ± 20% (20 nM, *p* = 0.036), 43 ± 47% (50 nM, *p* = 0.005), and 37 ± 38% (200 nM; *p* = 0.034; Figure 2A). Burst amplitude remained increased compared to time controls during washout at the 35-min time point by 45 ± 39% (50 nM, *p* < 0.001), 34 ± 37% (100 nM, *p* = 0.004), and 54 ± 54% (200 nM, *p* < 0.001; Figure 2A). Overall DAMGO concentration effects across the 15- and 35-min time points were observed for the 50 nM (*p* = 0.020) and 200 nM (*p* = 0.003) applications. There was no overall drug effect for the 100 nM application (*p* = 0.339) although data at the 35-min time point were significantly elevated compared to time controls. After an additional 40-min washout period (75-min time point), burst amplitude returned to baseline levels at all doses except for the 200 nM DAMGO application, which remained elevated at 40 ± 37% above baseline (*p* < 0.001 compared to time controls). The maximum percent increase in burst amplitude (occurring within the 15–60 min period after starting the DAMGO application) showed a wide range of responses for each DAMGO concentration, with a range up to a 146% increase for the 50 nM DAMGO (Figure 2B). The mean maximum burst amplitude increase was significant for the 50 nM (*p* = 0.013) and 200 nM (*p* < 0.001) DAMGO applications compared to time controls. There were no sex-dependent differences in the DAMGO-induced increases in the mean maximum burst amplitude (*p* = 0.172; *n* = 25 males, 33 females).

Baseline burst frequency was 9.8 ± 2.0 bursts/min for all preparations but was reduced by DAMGO at the 15-min time point (20 nM, *p* = 0.004; 50 nM, *p* < 0.001; 100 nM, *p* < 0.001; 200 nM, *p* < 0.001) and at the 35-min time point (50 nM, *p* < 0.001; 100 nM, *p* < 0.001; 200 nM, *p* < 0.001) compared to time controls, with significant overall DAMGO concentration effects (50 nM, *p* < 0.001; 100 nM, *p* < 0.001; 200 nM, *p* < 0.001; Figure 2C). Burst frequency returned to levels similar to time controls during the prolonged washout period except for the 200 nM DAMGO application which remained significantly depressed by 28 ± 14% (*p* = 0.007 compared to time controls). A complete cessation of motor activity for more than 10 min occurred during one 50 nM and five 200 nM DAMGO applications, showing that 200 nM DAMGO exerts an overriding respiratory depression in some preparations. Minute activity was decreased at the 15-min time point by 48 ± 32% (50 nM; *p* < 0.001), -61 ± 26% (100 nM; *p* < 0.001), and -85 ± 61% (200 nM; *p* < 0.001) compared to time controls (Figure 2D). For the 20 nM DAMGO application at the 15-min time point, there was no change in minute activity (Figure 2D) despite a significant increase in burst amplitude (Figure 2B) and a significant decrease in burst frequency (Figure 2C). Likewise, minute activity was not changed compared to time controls for the 50 nM (*p* = 0.697), 100 nM (*p* = 0.225), and 200 nM (*p* = 0.846) DAMGO applications at the 35-min time point despite significant increases in burst amplitude and decreases in burst frequency (Figure 2D). The lack of change in minute activity indicated that overall neuronal activity was preserved, but the features of the neural output were transformed into a lower frequency, higher amplitude rhythm. With respect to burst shape, baseline burst duration for all preparations was 1040 ± 200 ms and baseline burst rise time was 200 ± 90 ms, which indicates that the inspiratory burst shape was rapidly incrementing (e.g., Figure 1A). DAMGO did not alter the percent change in burst duration (*p* > 0.445 for overall DAMGO concentration effect) or percent change in burst rise time (*p* = 0.290 for overall DAMGO concentration effect) (data not shown).

To study potential explanations for the variability in the DAMGO-induced burst amplitude increase, the maximum change in burst amplitude was compared to specific characteristics of baseline activity. Linear regression analysis showed a positive correlation of the maximum change in burst amplitude with baseline burst frequency (*p* < 0.001, R^2^ = 0.428; Figures 3A,B) and baseline burst duration (*p* = 0.010, R^2^ = 0.154; Figure 3C), but not baseline burst peak percent (*p* = 0.732, R^2^ = 0.00297; Figure 3D). There was a weak significant negative correlation between maximum burst amplitude and the maximum percent frequency decrease (*p* = 0.015, R^2^ = 0.140; Figure 3E). Also, maximum burst frequency was not correlated with neonatal rat age in hours (*p* = 0.42, R^2^ = 0.0993; data not shown). These data show that preparations with relatively higher baseline burst frequencies and longer burst durations at baseline tended to have larger burst amplitude responses to DAMGO, and that the magnitude of DAMGO-induced increases in burst amplitude were inversely proportional to DAMGO-induced decreases in burst frequency.

**FIGURE 4:**
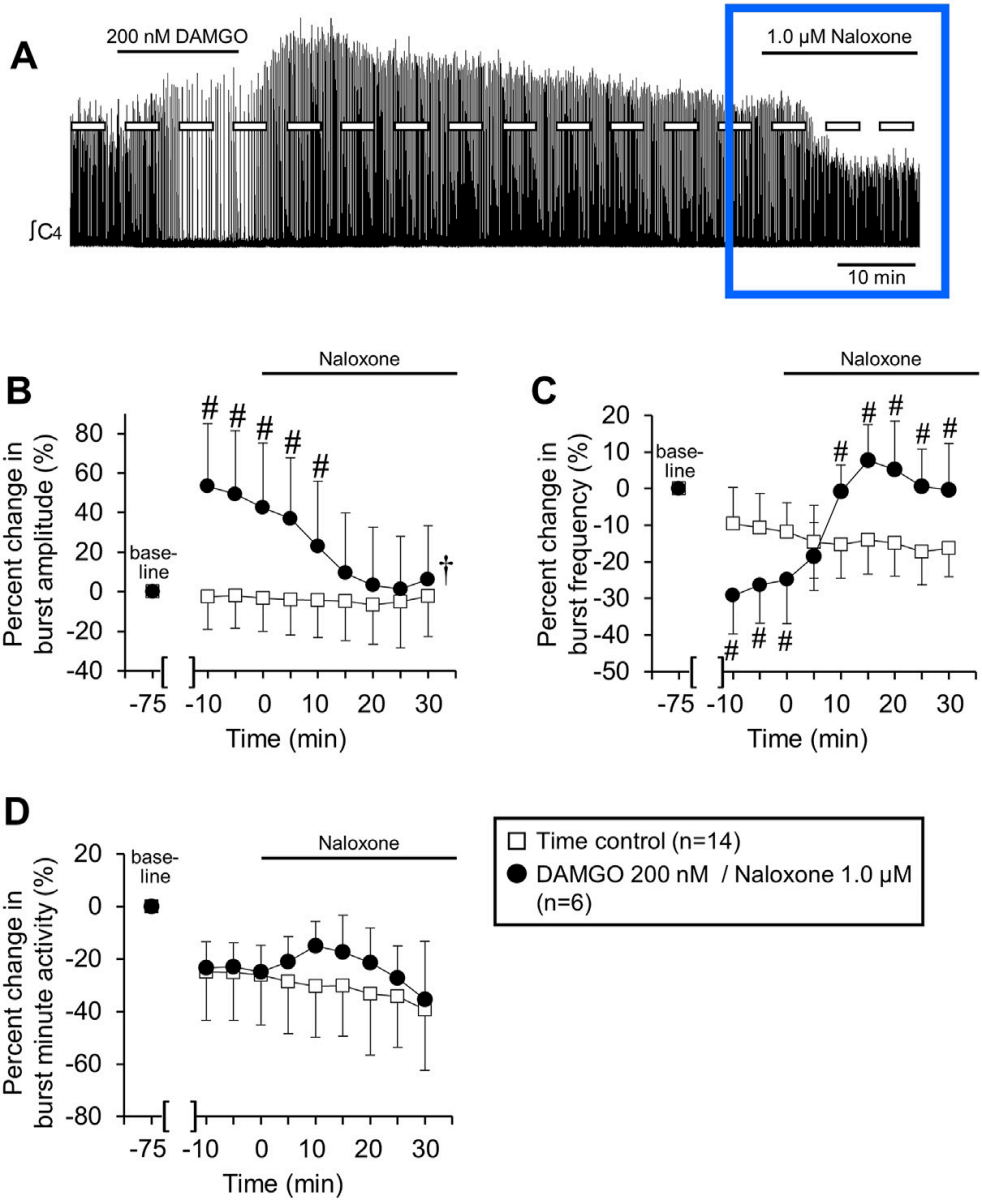
Naloxone reversed persistent DAMGO-induced effects on inspiratory burst amplitude and frequency. Six/10 brainstem spinal cord preparations exposed to 200 nM DAMGO (15 min) had a persistent increase in burst amplitude after 60 min of washout. Naloxone (1.0 µM) was bath-applied to test whether DAMGO effects could be reversed. **(A)** Compressed voltage trace showing baseline (left) and DAMGO-induced changes in burst amplitude and frequency that rapidly returned to baseline with naloxone application. The blue box highlights the part of voltage traces that were analyzed for the graphs below. **(B)** Naloxone application rapidly returned increased burst amplitude to time control levels. **(C)** Naloxone application rapidly increased burst frequency above time control levels. **(D)** Minute activity was not altered by naloxone application. The pound sign (#) indicates a significant pairwise comparison difference from time controls at that time point, and the dagger (†) indicates a significant overall DAMGO concentration effect. Data shown as mean ± SD. These data indicate that the persistent DAMGO-induced inspiratory motor burst increase was not likely due to neuroplasticity.

The persistent increase in burst amplitude after 60 min of washout for the 200 nM application (Figure 2A) suggested that DAMGO generated a type of respiratory neuroplasticity (i.e., long-lasting change in respiratory function that persists in the absence of the inducing stimulus). To test this hypothesis, naloxone (MOR competitive antagonist, 1.0 µM) was bath-applied to brainstem-spinal cord preparations that were exposed to 200 nM DAMGO and had a persistent increase in burst amplitude after 60 min of washout (*n* = 6; Figure 4A). Naloxone rapidly decreased inspiratory burst amplitude to near time control levels within 15 min (Figure 4B) and increased burst frequency to above time control levels within 10 min of application (Figure 4C). Minute activity was not altered from time controls during naloxone application (Figure 4D). In separate experiments, bath-applied naloxone (1.0 µM, 25 min) did not alter burst amplitude or frequency (*n* = 2, data not shown). The long-lasting increase in burst amplitude was likely not neuroplasticity, but instead due to persistent MOR activation.

### DAMGO-induced inspiratory burst amplitude increase not altered by cervical spinal midline lesion

**FIGURE 5:**
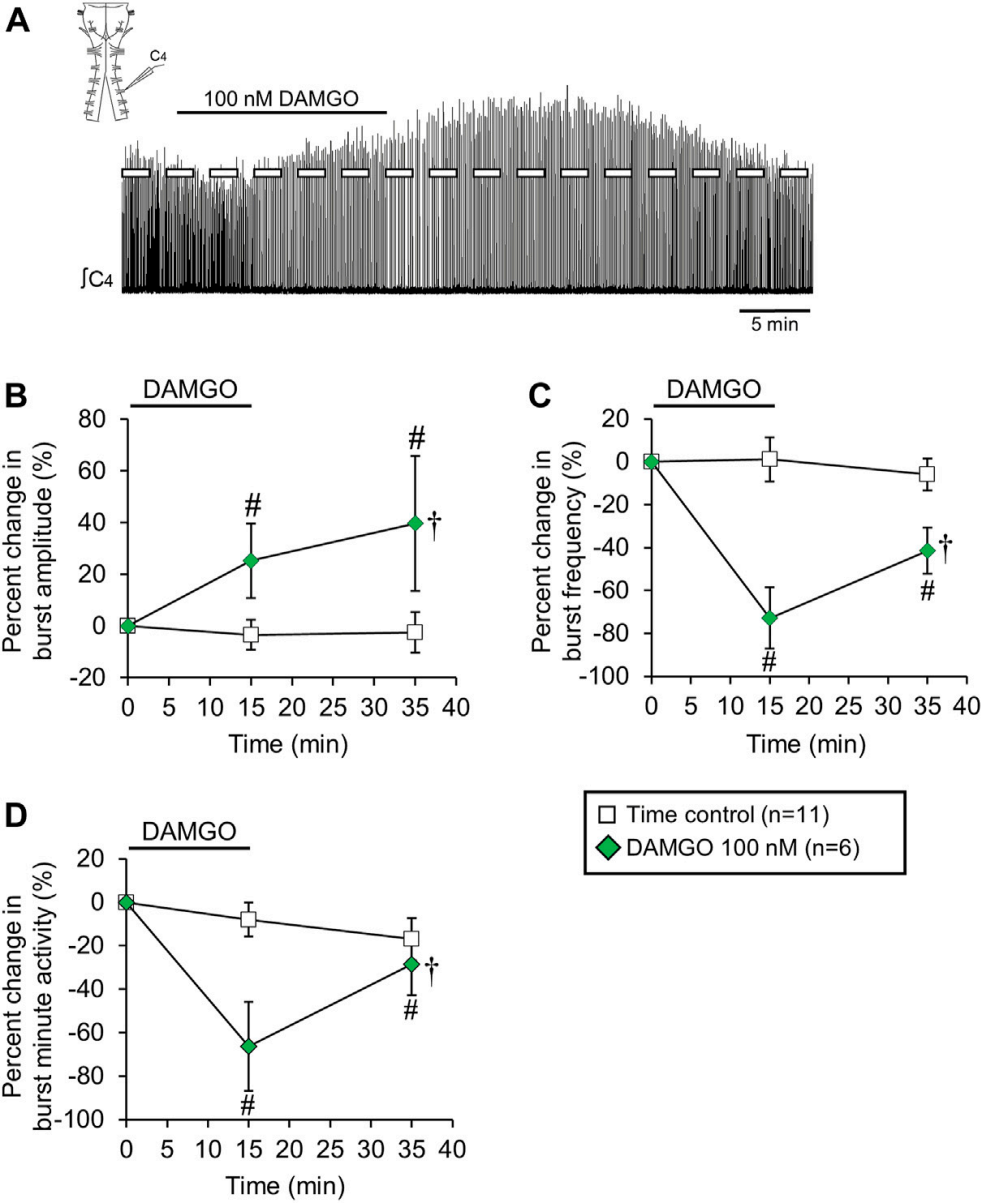
Cervical spinal midline-lesion did not alter the DAMGO-induced inspiratory burst amplitude increase. **(A)** Drawing of a brainstem-spinal cord preparation with the cervical spinal midline-lesion from C3 to C7 with a suction electrode attached at ventral cervical spinal root C4. At right is a sample voltage trace showing DAMGO-induced changes in burst amplitude and frequency. DAMGO (50–200 nM; green diamond symbols) increased burst amplitude **(B)** and decreased burst frequency **(C)** compared to time controls. **(D)** Minute activity was also decreased by DAMGO. The pound sign (#) indicates a significant pairwise comparison difference from time controls at that time point, and the dagger (†) indicates a significant overall DAMGO concentration effect. Data shown as mean ± SD. These data indicate that crossed bulbospinal pathways in the cervical spinal cord at C3-C7 were not necessary for the DAMGO-induced inspiratory burst amplitude increase.

Bulbospinal excitatory synaptic inputs to phrenic motoneurons cross over at the level of the phrenic motor nucleus in the cervical spinal cord. These crossed phrenic pathways are often silent but can be activated under different experimental conditions to increase inspiratory drive to phrenic motoneurons (termed the “crossed phrenic phenomenon”; Goshgarian, 2003). Because neonatal rats also express these crossed phrenic pathways (Zimmer and Goshgarian, 2005), the DAMGO-induced burst amplitude increase may be due to activation of these otherwise latent pathways. To test this possibility, brainstem-spinal cords were midline-lesioned at C3-C8 before recording inspiratory-related motor output on spinal C4 roots (Figure 5A). Cervical spinal midline-lesioned preparations were either untreated (time controls; *n* = 11) or exposed to 100 nM DAMGO (*n* = 6). Compared to time controls, DAMGO application increased burst amplitude compared to time controls by 25 ± 14% (*p* < 0.001) and 40 ± 26% (*p* < 0.001) at the 15-min and 35-min time points, respectively (*p* < 0.001 for overall DAMGO concentration effect; Figure 5B) and decreased baseline burst frequency (11.5 ± 1.9 bursts/min) by 68 ± 13% (*p* < 0.001) at the 15-min time point compared to time controls (*p* < 0.001 for overall DAMGO concentration effect; Figure 5C). Minute activity was decreased by 64 ± 22% (*p* < 0.001 compared to time controls) at the 15-min time point and by 31 ± 12% (*p* = 0.03 compared to time controls) at the 35-min time point (*p* < 0.001 for overall DAMGO concentration effect; Figure 5D). The cervical spinal midline-lesioned preparations had an increased baseline burst frequency compared to untreated preparations (11.5 ± 1.9 bursts/min vs. 8.9 ± 1.2 bursts/min; *p* = 0.003 for pairwise comparison), but baseline burst durations were similar for (untreated preparations = 1030 ± 140, lesioned preparations = 1070 ± 120 ms; *p* = 0.57 for pairwise comparison). Thus, the DAMGO-induced burst amplitude increase was expressed in spinally midline-lesioned brainstem-spinal cord preparations.

### DAMGO-induced inspiratory burst amplitude increase expressed in thoracic spinal cord

**FIGURE 6:**
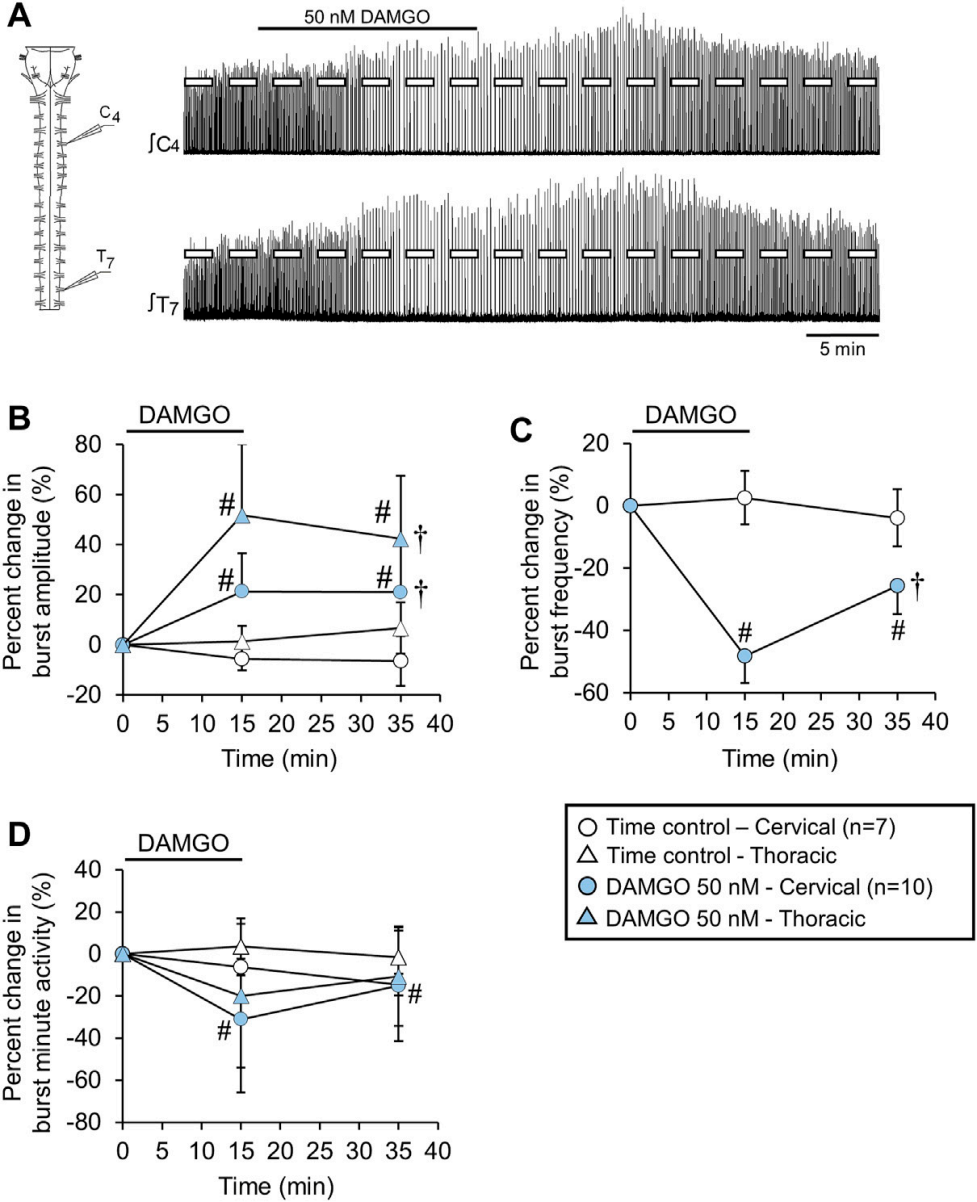
The DAMGO-induced inspiratory burst amplitude increase was expressed in motor output from the thoracic spinal cord. **(A)** Drawing of a brainstem-spinal cord preparation extended to thoracic spinal segment T8 with suction electrodes attached to ventral roots of spinal segments C4 and T7. At right are sample voltage traces showing inspiratory-related motor bursts in cervical (upper trace) and thoracic (lower trace) during a 50 nM DAMGO application. **(B)** Burst amplitude increased in both cervical (blue circles) and thoracic (blue triangles) spinal cord motor output. **(C)** Burst frequency was decreased by DAMGO. The decrease was identical for brainstem and spinal cord recordings (blue circles), while **(D)** minute activity was relatively unaltered by the DAMGO application except for the cervical motor output at the 15-min time point. The pound sign (#) indicates a significant pairwise comparison difference from time controls at that time point, and the dagger (†) indicates a significant overall DAMGO concentration effect. Data shown as mean ± SD. These data indicate that the inspiratory burst amplitude increase was not restricted to phrenic motoneurons in the cervical spinal cord.

The DAMGO-induced inspiratory burst amplitude increase may be expressed exclusively in phrenic motoneurons. If the burst amplitude increase is expressed in other spinal motoneuron pools, such as thoracic motoneurons driving intercostal muscles, this may suggest a common mechanism within the motor nuclei or a common source of increased drive from the brainstem. To test whether the DAMGO-induced inspiratory burst amplitude increase was also expressed in thoracic motoneurons driving intercostal muscles, brainstem-spinal cords were isolated that contained the thoracic spinal cord to spinal thoracic segments T4 or T8. Suction electrodes were attached to C4-C5 roots and T2-T7 roots. Brainstem-spinal cord preparations were either not treated (time controls; *n* = 7) or exposed to 50 nM DAMGO (*n* = 10, 15-min application). The DAMGO-induced inspiratory burst amplitude increase was observed simultaneously in the inspiratory-related motor output of both cervical and thoracic roots (Figure 6A). The percent increase in burst amplitude was larger in the thoracic motor output (e.g., 52 ± 28% thoracic compared to 21 ± 20% cervical at the 15-min time point; *p* < 0.001 for pairwise comparison) but both had significant overall DAMGO concentration effects (*p* = 0.001; Figure 6B). The magnitude of DAMGO-induced changes in burst amplitude for a given burst in the cervical spinal cord correlated strongly with the magnitude of DAMGO-induced changes in burst amplitude for the simultaneous burst in the thoracic spinal cord (*p* < 0.001; R^2^ = 0.919). Baseline burst frequency (12.0 ± 1.6 bursts/min) was decreased by 48 ± 20% (*p* < 0.001) and 26 ± 15% (*p* < 0.001) at the 15-min and 35-min time points compared to time controls, respectively (*p* < 0.001 for overall DAMGO concentration effect; Figure 6C). Minute activity was not altered in thoracic motor output at any time during or following the DAMGO application (*p* = 0.162 for overall DAMGO concentration effect), despite significant and reciprocal changes in inspiratory burst amplitude and frequency. Minute activity for the cervical motor output was decreased by 31 ± 35% (*p* < 0.001) and 15 ± 26% (*p* = 0.03) at the 15-min and 35-min time points compared to time controls, respectively, without a significant overall DAMGO concentration effect (*p* = 0.262; Figure 6D).

### Riluzole did not alter the DAMGO-induced inspiratory burst amplitude increase

**FIGURE 7:**
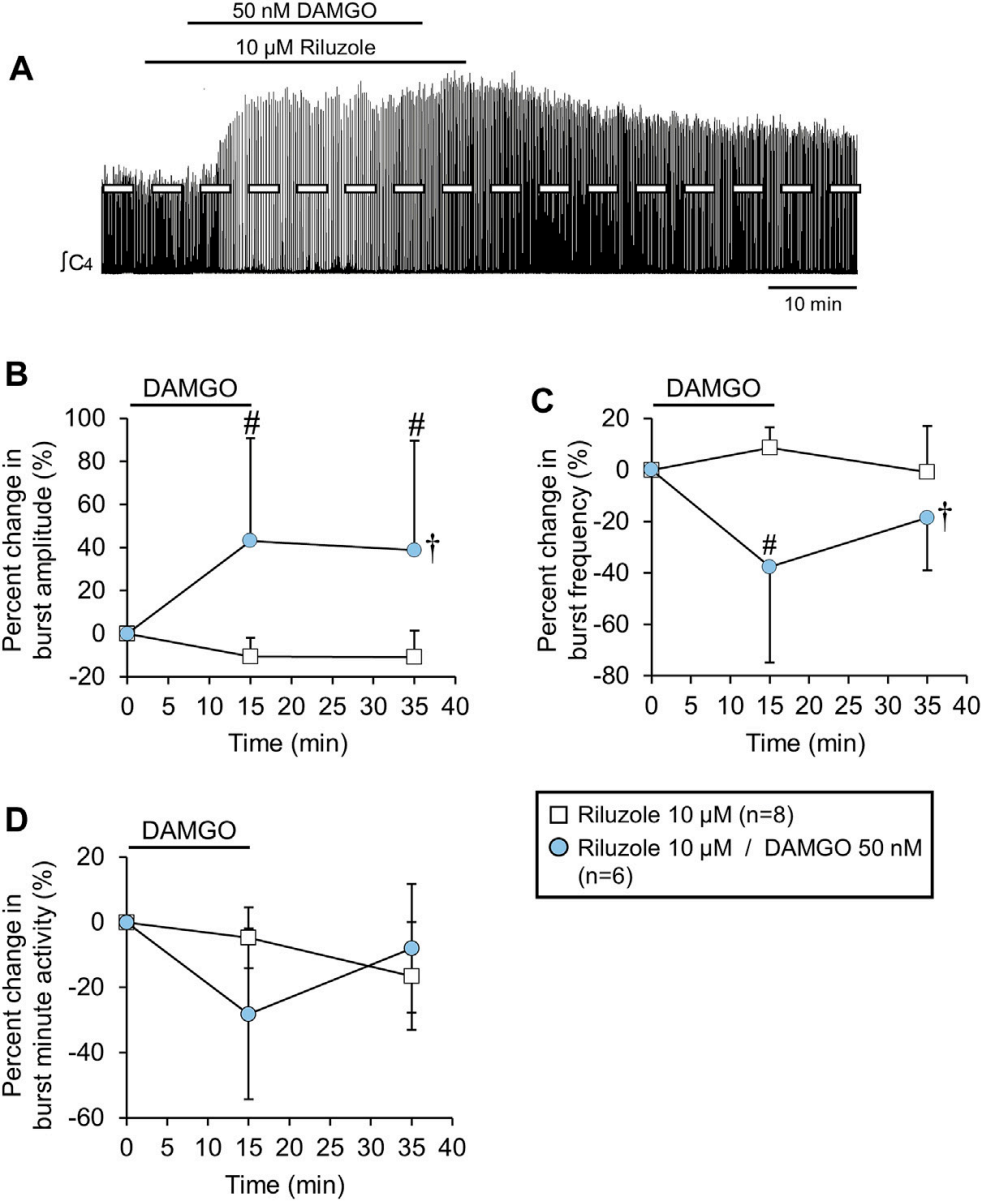
The DAMGO-induced burst amplitude increase was not altered by riluzole. **(A)** Voltage trace showing that DAMGO increased burst amplitude and decreased burst frequency during a background riluzole application. **(B)** Burst amplitude increased and **(C)** burst frequency decreased, while **(D)** minute activity was not altered (blue circles) compared to riluzole time controls (white squares). The pound sign (#) indicates a significant pairwise comparison difference from time controls at that time point, and the dagger (†) indicates a significant overall DAMGO concentration effect. Data shown as mean ± SD. These data suggest that the DAMGO-induced burst amplitude increase was not related to gasping since riluzole blocks persistent sodium currents associated with gasping.

Murine rhythmically-active medullary slices exposed to hypoxia shift from a “eupnic” motor pattern to a “gasping” motor pattern that is characterized by a slower rhythm, larger burst amplitude, and a shift in the preBotC burst shape to a rapidly incrementing/slowly decrementing shape (Lieske et al., 2000; Pena, 2009). The controversies with respect to whether neonatal rat brainstem-spinal cord preparations produce “eupnic” or “gasping” motor patterns is reviewed in Johnson et al. (2012). In the present study, the rapidly incrementing/slowly decrementing burst pattern (Figure 1A) at baseline increased in amplitude with DAMGO administration, suggesting the contribution of the gasping mechanism. Gasping requires a persistent sodium current that is sensitive to riluzole (Pena and Aguileta, 2007) and bath-applied 10 µM riluzole is sufficient to block ∼85% of the persistent sodium current in rat *in vitro* preparations (Del Negro et al., 2002). To test whether the DAMGO-induced inspiratory burst amplitude increase required activation of persistent sodium currents, DAMGO was bath-applied (50 nM, 15-min) during a background bath-application of riluzole (10 μM, applied 5 min before, during, and 5 min after the DAMGO application) to brainstem-spinal cord preparations (n = 6; Figure 7A). Riluzole by itself was bath-applied (10 μM, 25-min) to separate brainstem-spinal cord preparations (n = 8) to control for potential time-dependent riluzole effects. When DAMGO was co-applied with riluzole, burst amplitude increased by 43 ± 48% (*p* = 0.003) and 39 ± 51% (*p* = 0.002) at the 15-min and 35-min time points compared to time controls, respectively (*p* = 0.012 for overall DAMGO concentration effect; Figure 7B). Burst frequency decreased (baseline frequency = 12.5 ± 2.0 bursts/min) by 38 ± 37% (*p* < 0.001) and 19 ± 20% (*p* = 0.039) at the 15-min and 35-min time points compared to time controls, respectively (*p* = 0.013 for drug effect; Figure 7C). Minute activity was not altered (*p* = 0.412 for overall DAMGO concentration effect; Figure 7D). Thus, the effects of 50 nM DAMGO were similar with respect to the amplitude increase and frequency decrease for preparations without and with the background riluzole application.

### DAMGO-induced inspiratory burst amplitude increase due to brainstem MOR activation

**FIGURE 8:**
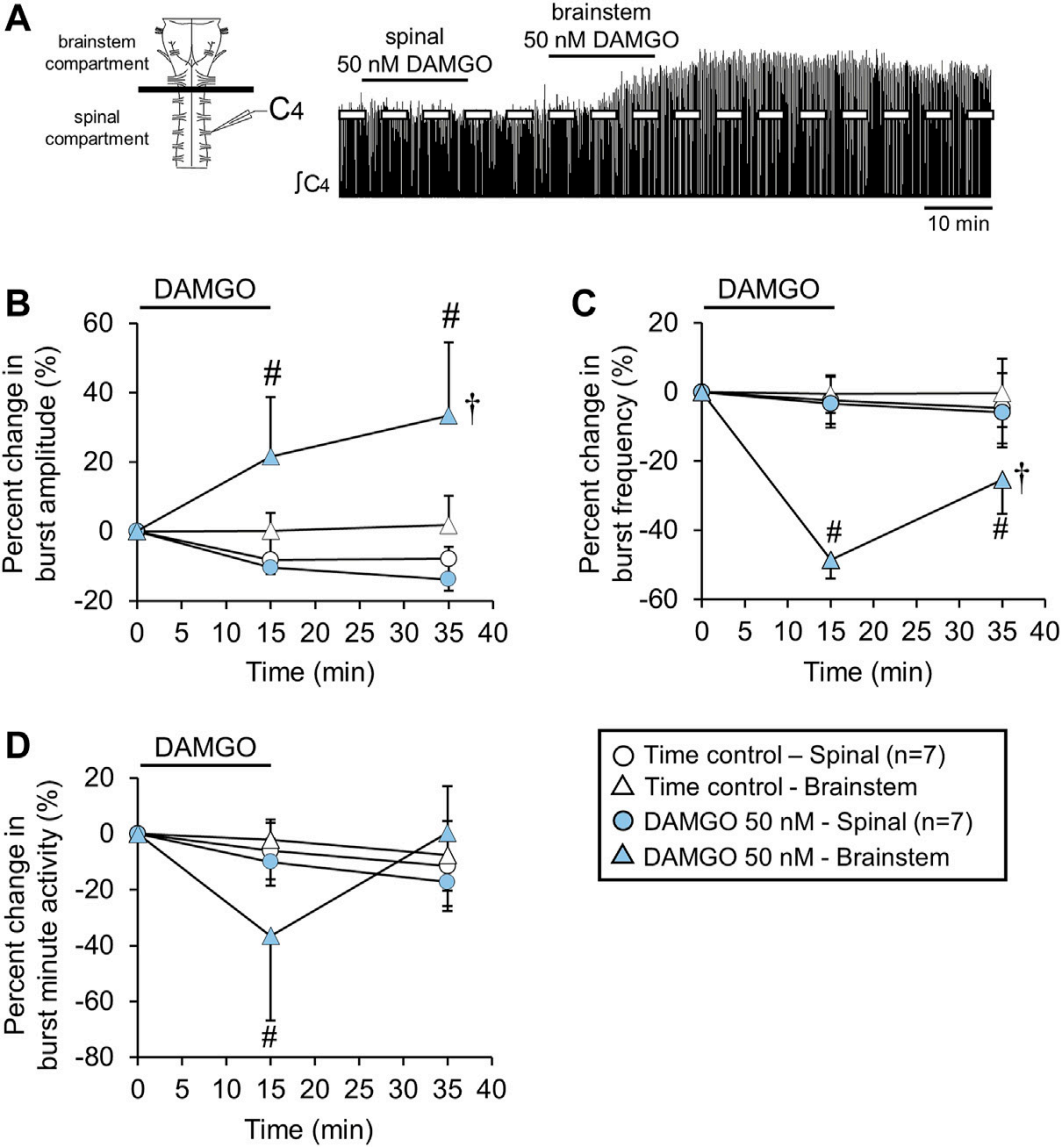
The DAMGO-induced burst amplitude increase was due to brainstem MOR activation. **(A)** Drawing of brainstem-spinal cord preparation with a plastic and petroleum jelly barrier that separated the recording chamber into separately perfused brainstem and spinal compartments. At right is a voltage trace showing the effects of bath-applied DAMGO (50 nM) in the spinal compartment first, followed by a similar bath-application in the brainstem compartment. **(B)** The burst amplitude increase occurred only when DAMGO was bath-applied in the brainstem compartment. Data for time controls and DAMGO application experiments are indicated by the white and blue symbols, respectively (spinal application = circles; brainstem application = triangles). **(C)** Burst frequency depression was only observed when DAMGO was bath-applied to the brainstem compartment. **(D)** Minute activity decreased at the 15-min time point when DAMGO was applied to the brainstem and not the spinal cord. Otherwise, there were no overall DAMGO concentration effect of DAMGO on minute activity. The pound sign (#) indicates a significant pairwise comparison difference from time controls at that time point, and the dagger (†) indicates a significant overall DAMGO concentration effect. Data shown as mean ± SD.

The DAMGO-induced increase in burst amplitude could be due to MOR activation in the spinal cord or medulla, or both. To test whether the DAMGO-induced inspiratory burst amplitude increase was due to brainstem or spinal mechanisms, the recording chamber was partitioned into separate brainstem and spinal compartments that were perfused with different aCSF solutions (Figure 8A). Time control brainstem-spinal cord preparations (*n* = 7) were placed in a partitioned recording chamber and no DAMGO was applied. For the treated brainstem-spinal cord preparations (*n* = 7), DAMGO (50 nM) was first bath-applied to the spinal cord compartment for 15 min, followed by a 15-min recovery period, and then DAMGO (50 nM) was bath-applied to the brainstem compartment. For data comparison between spinal and brainstem DAMGO applications, data were aligned with time 0 min as the time before each DAMGO application. Burst amplitude was unaltered with spinal DAMGO application (*p* = 0.321 for overall DAMGO concentraton effect) whereas burst amplitude increased by 22 ± 17% (*p* = 0.002) and by 34 ± 19% (*p* < 0.001) at the 15-min and 35-min time points compared to time controls, respectively, with the brainstem DAMGO application (*p* = 0.004 for overall DAMGO concentration effect; Figure 8B). Spinal DAMGO application did not alter burst frequency (*p* = 0.812 for overall DAMGO concentration effect), but brainstem DAMGO application decreased burst baseline frequency (8.5 ± 1.7 bursts/min) by 49 ± 23% (*p* < 0.001) and by 25 ± 13% (*p* < 0.001) at the 15-min and 35-min time points compared to time controls, respectively (*p* < 0.001 for overall DAMGO concentration effect; Figure 8C). Spinal DAMGO application did not alter minute activity (*p* = 0.374 for overall DAMGO concentration effect), but brainstem DAMGO application decreased minute activity by 36 ± 30% (*p* < 0.001 compared to time controls) at the 15-min time point but not at the 35-min time point (0 ± 21%, *p* = 0.966 compared to time controls; Figure 8D). DAMGO (50 nM) application to the brainstem compartment without a prior spinal DAMGO application also induced the burst amplitude increase (*n* = 3; data not shown), which ruled out a potential “priming effect” that might occur with spinal DAMGO application preceding brainstem application. These data suggest that the DAMGO-induced inspiratory burst amplitude increase was due to MOR activation in the brainstem.

## Discussion

The main finding was that brainstem MOR activation transformed the respiratory network to produce a motor pattern with an increased burst amplitude and decreased frequency. In several experiments, this transformation represented little change in overall motor output, especially at the lower DAMGO concentrations (e.g., 20 nM). DAMGO application induced an inspiratory burst amplitude increase and frequency depression in a manner that represented a form of neuromodulation, and not neuroplasticity, because naloxone rapidly reversed the DAMGO-induced effects. The DAMGO-induced inspiratory burst amplitude increase did not require crossed spinal connections between C3 and C7 and was resistant to riluzole pre-treatment, which suggests that these effects were likely not related to gasping. The DAMGO-induced inspiratory burst amplitude increase was expressed in thoracic spinal ventral roots as well as cervical spinal roots, suggesting a common source for the burst amplitude increase. Split-bath experiments revealed that this DAMGO-induced neuromodulation occurred in the brainstem and not the spinal cord, suggesting that the transformation in respiratory motor output happened in pre-motor or respiratory rhythm-generating areas and not in respiratory motor pools.

### MOR-dependent effects on respiratory motor control in neonates

Many factors influence how MOR activation modulates the neural control of breathing, such as species (Stucke et al., 2008; Mustapic et al., 2010; Cinelli et al., 2020), age (Willmann et al., 2009), and physiological state (Holaday, 1983; Lalley, 2008; Dong et al., 2020), as well as experimental factors such as concentration (Greer et al., 1995; Mutolo et al., 2007), time course, and mode of drug application (Czapla et al., 2000). In neonatal rats, MOR activation at higher doses is generally associated with reduction in ventilation and breathing frequency, which are hallmarks of opioid-induced respiratory depression (Greer et al., 1995; Colman and Miller, 2001). MOR agonist drugs (dermorphin and fentanyl) decrease minute volume and breathing frequency in neonatal (P2-P8) rats, but there is also a striking increase in tidal volume that is hypothesized to be compensatory and related to gasping (Colman and Miller, 2001). These findings *in vivo* using plethysmography are similar to the transformation in respiratory motor pattern observed in this study, but we suggest that gasping mechanisms are not involved (see below).

With respect to neonatal rodent *in vitro* preparations, responses to MOR activation depend on which anatomical structures are included in the preparation. For example, the frequency of inspiratory motor bursts on C4 spinal roots increases following bath-applied DAMGO in brainstem-spinal cord preparations containing the pons (Tanabe et al., 2005). Coincident with the DAMGO-dependent frequency increase, there is increased excitability of respiratory-related neurons in the Kolliker-Fuse region in the rostral pons (Kobayashi et al., 2005) and a subpopulation of respiratory-related neurons in the pFRG/RTN region in the caudal pons (Tanabe et al., 2005). In contrast, MOR activation decreases burst frequency in brainstem-spinal cord preparations lacking an intact pons (e.g., Greer et al., 1995; Ballanyi et al., 1997; Takita et al., 1997; Takita et al., 1998). Furthermore, in these preparations, the activity of pre-I neurons in the pFRG/RTN region is not altered by bath-applied MOR agonist drugs (Takeda et al., 2001; Janczewski et al., 2002; Mellen et al., 2003). Interestingly, Tanabe et al. (2005) state that burst amplitude decreased with bath-applied 200 nM DAMGO in similar neonatal rat brainstem-spinal cord preparations lacking the pons, a finding that is direct contrast to this study. This discrepancy could be due to the precise level of the rostral border of the preparations, differences in rat strains, or experimental protocol. For example, DAMGO was applied for ∼10 min in Tanabe et al. (2005) and there is no mention of a washout period. In our study, 200 nM DAMGO could abolish the respiratory rhythm during DAMGO application and the peak burst amplitude increase may not be observed until 10–15 min into the washout period.

In the present study, bath-applied DAMGO (10–200 nM) resulted in variable but dramatic effects (>80% increase bilaterally in some preparations) on inspiratory burst amplitude. There was, however, considerable variability in the maximum change in burst amplitude (Figure 2B) such that the 50 nM DAMGO application resulted in burst amplitude changes ranging from 3% to 146% increase. Some variability was likely due to the “state” of the preparation prior to drug application because the magnitude of the burst amplitude increase was positively correlated with baseline burst frequency and duration. Preparations with a higher baseline burst frequency and longer burst duration tended to have larger increases in burst amplitude following DAMGO application. The factors that determine the “state” of the preparation at the time of DAMGO application are not well understood, given the various limitations of brainstem-spinal cord preparations (Johnson et al., 2012). Also, until the mechanism underlying the MOR-dependent inspiratory burst amplitude increase is known, it is difficult to hypothesize why some preparations are more likely to exhibit a larger increase in burst amplitude.

### Transformation of respiratory motor output by MOR activation

At certain DAMGO concentrations and times during drug washout, the simultaneous increase in burst amplitude and the decrease in burst frequency were balanced such that there was no significant change in minute activity. For example, there was no change in minute activity during DAMGO (20 nM) application even though there was a significant burst amplitude increase and frequency decrease. Likewise, after 20 min of drug washout, there were no changes in minute activity for the 50–200 nM DAMGO applications although there were still significant increases in burst amplitude and decreases in burst frequency. The observation that MOR activation produced increased burst amplitude and decreased burst frequency suggests that low levels of MOR activation transform the respiratory network to produce a different pattern of inspiratory motor output while maintaining overall minute activity. Although not verified experimentally, inspiratory burst amplitude on C4-C5 ventral spinal roots produced by *in vitro* preparations is likely related to tidal volume. For intact neonatal rats, low level MOR activation may result in increased tidal volume and decreased breathing frequency while still maintaining overall ventilation. Such a transformation may not be associated with a specific respiratory motor pattern per se (e.g., sighing, coughing), but rather contribute to various patterns where this transformation is important for maintaining blood-gas homeostasis.

### Localization of the DAMGO-induced inspiratory burst amplitude increase

The inspiratory burst amplitude increase was expressed in thoracic spinal motor output and tightly correlated with changes in cervical spinal motor output. This suggested that the mechanism for the burst amplitude increase was occurring upstream from a common source in the medulla. The split-bath experiments showed that DAMGO applied to the spinal cord compartment had no effect on inspiratory burst amplitude, whereas DAMGO application to the brainstem compartment induced the inspiratory burst amplitude increase. These experiments rule out the involvement of the phrenic or thoracic intercostal motor pools and indicate that MOR activation in the brainstem is required for the expression of the inspiratory burst amplitude increase. The experiments also demonstrate that phrenic motoneuron activity is not directly depressed by the administrated DAMGO concentrations. In decerebrate rabbits, naloxone injection into the respiratory rhythm generator completely prevented respiratory rate depression from high intravenous remifentanil doses, however, remifentanil continued to decrease peak phrenic motor activity (Palkovic et al., 2022). Our study suggests that this decrease was not due to direct depression of phrenic motoneurons but rather of excitatory drive to these neurons. Given the abundant MOR expression in the neonatal rat medulla (Xia and Haddad, 1991) and within the preBotzinger Complex (preBotC; Gray et al., 1999), bath-applied DAMGO at low concentrations likely activated MORs in multiple medullary regions with the net effect resulting in the inspiratory burst amplitude increase. We hypothesize that MOR activation in the preBotC region is causing the increased burst amplitude because microinjection of DAMGO into the preBotC of rhythmically active medullary slices appears to increase hypoglossal motor burst amplitude (Johnson et al., 1996). Low level activation of MORs on preBotC rhythm-generating neurons and pre-motoneurons may reconfigure the respiratory network to produce the inspiratory burst amplitude increase.

### Increased burst amplitude was not due to neuroplasticity, crossed spinal pathways, or gasping

At most DAMGO concentrations, the inspiratory burst amplitude increase was reversed within an hour of drug washout. DAMGO application at 200 nM, however, was a notable exception because burst amplitude was still increased by 40 ± 4% after 60 min of washout. This persistent burst amplitude increase did not appear to be a type of neuroplasticity because naloxone rapidly reversed the burst amplitude increase and frequency decrease. If neuroplasticity was induced, inspiratory burst amplitude increase would likely still be expressed because the mechanisms maintaining increased burst amplitude would be downstream in the signaling pathway following MOR activation. It is more likely that the persistent amplitude increase with 200 nM DAMGO was due to residual DAMGO in the recording dish during the washout period. DAMGO is very stable and has a long half-life (∼15 h) in brain homogenates (Van Dorpe et al., 2010). Since dye injected into the bath is readily cleared completely within 5 min (data not shown) given the chamber volume and flow rate, DAMGO could have been retained within the neural tissue itself and thereby caused a low-level persistent MOR activation during washout.

In neonatal rats, bulbospinal axons descend in the spinal cord and crossover within the phrenic motor nucleus (Zimmer and Goshgarian, 2005). To test whether these (potentially silent) excitatory synaptic inputs were recruited during the expression of the burst amplitude increase, the spinal cord was midline-lesioned at C3-C7 to disrupt these pathways. The magnitude of the inspiratory burst amplitude increase was similar with bath-applied 100 nM DAMGO (Figures 2A, 5A) for intact and cervical spinal midline-lesioned brainstem-spinal cord preparations. Thus, we conclude that crossed phrenic pathways are likely not involved in increased burst amplitude following DAMGO application.

In this study, the pattern of the DAMGO-induced inspiratory burst increase resembled the low frequency, large amplitude motor bursts that are observed when neonatal rodent *in vitro* preparations are exposed to hypoxia (Lieske et al., 2000; Pena, 2009). Since the burst pattern of the brainstem-spinal cord preparations is rapidly-incrementing, similar to the *in vitro* gasping pattern (Lieske et al., 2000), the gasping feature that involves inspiratory burst transformation cannot be assessed in this study. Nevertheless, the significant increase in burst amplitude with DAMGO administration raised the question whether this could reflect a gasping mechanism. Bath-applied DAMGO, however, still induced the inspiratory burst amplitude increase during a background riluzole application designed to block nearly all of the persistent sodium current underlying gasping *in vitro* (Del Negro et al., 2005; Pena and Aguileta, 2007). Furthermore, DAMGO application in this study did not alter motor burst shape (e.g., burst duration or burst rise time) whereas motor burst shape shifts to a rapid-onset, shorter duration burst when rhythmic *in vitro* preparations are exposed to hypoxia and exhibit gasping (Lieske et al., 2000; Barnes et al., 2007). Thus, the DAMGO-induced inspiratory burst amplitude increase and slowing of burst frequency is not likely to be related to gasping.

### Potential mechanisms underlying the burst amplitude increase

At the cellular and synaptic level, MOR activation initiates a signaling pathway through canonical Gi/o-protein pathways to inhibit calcium channels, inhibit adenylyl cyclase (reducing [cAMP]), inhibit hyperpolarization-activated cation currents (HCN), and activate G-protein-activated inward rectifier potassium channels (GIRK) (Bateman and Levitt, 2021; Ramirez et al., 2021). The burst frequency decrease is hypothesized to be due to DAMGO-dependent decrease in “burstlet” frequency within the preBotC, which reduces the frequency of respiratory-related motor bursts produced by rhythmically active medullary slices (Sun et al., 2019). In contrast, it is not known whether any of these mechanisms play a role in the induction of the DAMGO-dependent inspiratory burst amplitude increase. One possible explanation is MOR-induced disinhibition, whereby MOR activation inhibits a population of inhibitory neurons within the medulla (*e.g.*, *via* potassium channel activation on presynaptic terminals containing GABA), which then act to increase excitatory synaptic drive within the respiratory control system. For example, in midbrain ventral tegmental neurons, MOR activation inhibits GABA release presynaptically, *via* two known signaling pathways (Zhang et al., 2015). Another possible explanation is that MOR activation directly causes postsynaptic excitation (Margolis et al., 2014) or augments postsynaptic responses to NMDA application (Martin et al., 1997). Finally, non-neuronal mechanisms may be involved such as MOR activation causing astrocytes to release glutamate (Nam et al., 2021) to excite respiratory-related neurons and increase burst amplitude. Additional studies are required to determine whether neuron-neuron or neuron-astrocyte communication underlies the MOR-dependent transformation of respiratory motor output.

The different time courses suggest distinct cellular and synaptic mechanisms underlying the DAMGO-induced burst amplitude increase and frequency decrease because the burst amplitude increase was often observed at the 35-min time point while the frequency decrease was resolving back towards levels in time controls. For example, burst frequency returned back to near baseline levels shortly after the DAMGO application was terminated while the burst amplitude increase was still expressed (Figure 1B, voltage traces for 100 nM and 200 nM DAMGO applications). Similar results are shown in panels B and C for Figures 5–8, where the burst amplitude increase is similar at the 15-min and 35-min time points whereas burst frequency is changing back towards time control levels. By testing the different specific mechanisms described above, it may be possible to experimentally dissociate the burst amplitude increase from the burst frequency decrease. Alternatively, the different time courses of the amplitude and frequency effects may be due, in part, to the experimental conditions whereby DAMGO diffused into the tissue to activate multiple signaling pathways at different tissue depths, thereby affecting the time course of the drug response and the effects of drug washout.

## Methodological considerations

Isolated *in vitro* preparations produce respiratory-related motor output that is variable from preparation to preparation due to the age and species of the animal, the size and location of brain tissue isolated, and intrinsic properties of respiratory neurons that can be biased with respect to excitability (Burgraff et al., 2021). This is especially true for isolated *in vitro* brainstem-spinal cord preparations from neonatal rodents where the presence or absence of the pons is a major determinant of respiratory motor burst frequency (e.g., see Tanabe et al., 2005). In this study, burst frequency was variable as shown in Figure 3B and the DAMGO-induced burst amplitude increase was larger in preparations with a higher baseline burst frequency. Thus, experimental conditions may contribute to the variability in burst frequency and the DAMGO-induced burst amplitude increase. Also, comparison with other studies is problematic because of key differences in the experimental approach. For example, no burst amplitude increase was observed in a semi-intact *in situ* preparation (Saunders and Levitt, 2020), but the rostral transverse cut was at the collicular level (not pontomedullary level), fentanyl was the drug tested (not DAMGO), and the fentanyl was delivered systemically (not bath-applied). The efficacy and specificity for MORs vary between the common opioid drugs, such as DAMGO and fentanyl.

Finally, are the DAMGO concentrations used in this study similar to concentrations of endogenously released opioid peptides in brainstem respiratory neural circuits? Electron microscopy shows that there are examples of direct contact of endogenous opioid (endomorphin-2) containing presynaptic terminals in close apposition to MOR-expressing preBotC neurons (Qi et al., 2017). This suggests that direct synaptic transmission is a prominent mechanism for MOR activation in the preBotC, but volume transmission may be possible. Within synaptic clefts, the concentration of a neurotransmitter depends on the amount of peptide released, volume of the fluid in the cleft, rate of diffusion, and the rate of peptide metabolism and reuptake (Scimemi and Beato, 2009). Also, peptide release appears to require higher action potential firing frequencies to induce release (Iverfeldt et al., 1989). The complexities of synaptic transmission and the challenges of quantifying endogenous peptide levels with current techniques precludes the ability to interpret the results of this study that used low nanomolar DAMGO concentrations. We propose that the neuromodulatory effects on respiratory motor output at the low opioid concentrations become masked at higher opioid concentrations that cause marked respiratory depression and prolonged apneas.

### Physiological significance

The physiological context for release of endogenous MOR agonists is not clear. Likewise, the overall outcome of low levels of MOR activation on respiratory motor control in intact neonatal rodents is not well understood. One hypothesis is that relatively high endogenous opioid levels at birth excite and maintain rhythmogenic neuronal activity in the pFRG/RTN region, which then synaptically drives neurons in the preBotC (inspiratory rhythm generator) to maintain breathing and reduce apneas (Janczewski and Feldman, 2006; Oku et al., 2007). Alternatively, MOR activation may play a role in a complex “push-pull” neuromodulatory system that regulates and fine-tunes respiratory responses in neonates during development (Doi and Ramirez, 2008). Synaptic varicosities containing endogenous opioids, such as endomorphin-2 or enkephalin, are in close apposition to preBotC neurons immunopositive for neurokinin-1 receptors and MORs (Liu et al., 2004; Qi et al., 2017). Also, endomorphin-2 is colocalized with substance P in some presynaptic terminals, but not with presynaptic terminals containing GABA or glutamate (Qi et al., 2017). In contrast, enkephalin is colocalized within presynaptic terminals synthesizing GABA or glutamate (Liu et al., 2004). Synapses tend to release mostly an amino acid neurotransmitter when stimulated at low action potential frequencies, whereas amino acids and peptides are released together when trains of high frequency action potentials stimulate the synapse (Tallent, 2008). Thus, depending on the physiological context, the ratio of inhibitory receptor activation (by endogenous opioids, somatostatin, glycine, GABA) to excitatory receptor activation (glutamate, substance P) may be the key to shifting the respiratory network to a different state while stabilizing breathing (Doi and Ramirez, 2008). This study reveals a potentially novel form of respiratory neuromodulation elicited by low level MOR activation on respiratory-related neurons that can transform the pattern of motor activity without altering overall drive to respiratory muscles.

## Data Availability

The raw data supporting the conclusions of this article will be made available by the authors, without undue reservation.
